# Female football players’ key physical qualities: playing-position specific comparison between national-team selected and non-selected players

**DOI:** 10.5114/biolsport.2025.146780

**Published:** 2025-01-24

**Authors:** Eero Heikki Johannes Savolainen, Johanna Kaarina Ihalainen, Kristoffer Weckström, Tomi Vänttinen, Simon Walker

**Affiliations:** 1 Faculty of Sport and Health Sciences, University of Jyväskylä, Finland; 2 Finnish Institute of High Performance Sport KIHU, Jyväskylä, Finland; 3 Finnish Football Association, Helsinki, Finland; 4 Neuromuscular Research Center, University of Jyväskylä, Finland

**Keywords:** Soccer, Women, Speed, Power, Endurance

## Abstract

This study investigated 1) possible differences in physical qualities between national team selected and non-selected female football players specific to playing position and 2) possible between-position differences in physical qualities specific to competition level (national team selected and non-selected players separately). One-hundred eight-six female players from Finland’s senior and youth national teams (n = 85) and Finland’s national league teams (n = 101) participated in this cross-sectional study. The following field tests were used to characterize physical qualities of the players: 30-meter sprint test with 10-meter split time for speed, countermovement jump (CMJ) for lower-body power, and 1200-meter shuttle running test (i.e. Bronco test) was used to calculate maximal aerobic speed (MAS). National team selected central- and wide-defenders and central-midfielders were faster (g = 0.74–0.94, p = 0.042–0.007) in 10-meter sprint-time and central-midfielders and forwards were faster in 30-meter sprint-time (g = 0.73–0.81, p = 0.047–0.033) compared to non-selected counterparts. Selected wide-midfielders jumped higher in CMJ (g = 0.72, p = 0.048) than non-selected counterparts. Selected central- and wide-midfielders and forwards had higher MAS (g = 0.63–1.68, p = 0.037–0.001) than non-selected counterparts. Between-position analysis revealed that selected wide-midfielders reached higher CMJ height than central-midfielders and achieved higher MAS than central-defenders. Non-selected wide-defenders were faster for 30-meter sprint time than central-defenders, central-midfielders, and forwards. Physical qualities, especially speed, differentiate national team selected players from non-selected players. Between-position differences varied between groups, but players with better physical qualities played in wide-positions in both groups.

## INTRODUCTION

Previous studies have shown that higher-level female football players have better physical qualities in speed [1], strength [1–3] and endurance [2–5] compared to lower-level players, in general. However, current evidence suggests that physical qualities do not differentiate national team selected players from non-selected players when comparisons are made to elite-level players and specific to playing position [6]. Physical qualities are vital in football because they are highly associated to high-intensity running during matches [7] and the amount of high-intensity running is a determining factor between competition levels [8]. Match demands are shaped by several factors, which should be kept in mind when interpretating match running data [9]. High-intensity running (19–23 km/h) and sprint distance (> 23 km/h) covered during the match increased 15% and 29%, respectively, from the FIFA world cup 2015 to 2019 [10]. Although different tracking technology was used in the 2023 World Cup, which limits longitudinal benchmarking, sprint distance increased as a proportion of total distance in 2023 from 2019 (1.81% vs 1.71%) [9]. These findings demonstrate the trend of increasing match demands at elite-level and that the role of physical qualities could be even more important in the future.

Several studies have shown that match demands differ significantly between playing positions [11, 12]. Thus, it would be logical that players’ physical qualities also differ between playing positions. However, previous studies have shown mixed findings regarding differences in physical qualities between playing positions. Typically, studies have shown that outfield players have better physical qualities compared to goalkeepers, while findings between outfield positions have varied between studies [1, 4, 5, 13–15]. One clear limitation in almost all previous studies is that central- and wide-defenders and midfielders have been combined into the same groups (typically because of a small sample size), even though these players have different tactical roles within the match and that several studies have shown differences in match demands between wide- and central-positions [11, 12]. Bradley et al., [5] classified elite-level players into central- and wide-defenders and midfielders and found that wide-midfielders performed significantly better in Yo-Yo intermittent endurance test 2 compared to central-defenders and forwards. Conversely, Vagle et al., [15] used a similar classification for playing positions to national level players in Norwegian premier league but did not find differences in strength, power, sprint speed or agility test results between outfield playing positions. Based on these observations, it is possible that position-specific differences are only observable in the highest level of competition, whereas sub-elite players’ physical qualities may not differentiate between outfield playing position as much. Since match demands [11, 12] and physical qualities, at least at elite-level, [5] vary between positions it is important to compare national team selected players’ physical qualities to non-selected players specific to playing position.

To the authors’ knowledge, only one study has currently investigated this issue with USA’s National Women’s Soccer League (NWSL) national team selected and non-selected players [6]. They found moderate-to-large effect sizes for speed, endurance and jump test results in favor of national team selected attacking midfielders, defensive midfielders, goalkeepers, and wide midfielders, but the only statistically significant difference was national team selected defensive midfielders’ better performance in speed test compared to non-selected counterparts [6]. Thus, they concluded that physical qualities generally do not distinguish between competition levels in elite-level football [6].

NWSL is a full professional league [16] and all players are elite-level regardless of national team status. To the authors’ knowledge there are no studies that have investigated whether physical qualities differentiate national team selected from non-selected sub-elite level counterparts specific to playing position. Thus, the requirement of physical qualities for sub-elite players to progress to a higher level in their career should be clarified. Therefore, the aims of this study were to: 1) investigate possible differences in physical qualities between national team selected and non-selected sub-elite level players specific to playing position and 2) investigate possible between-positions differences in physical qualities specific to competition level.

## MATERIALS AND METHODS

### Participants, ethics and consent

One-hundred and eighty-six female football players from seven (out of ten) teams from Finland’s premier national league (n = 101, age 21 ± 4) and from Finland’s senior- (n = 15, age 24 ± 3), under 23-(n = 18, age 21 ± 2), under 19- (n = 28, age 18 ± 1) and under 17-year-old (n = 24, age 16 ± 0) national teams voluntarily participated to this study. Those teams from the national league that did not participate one team finished in top-table, one in mid-table and one was promoted from the lower division in the previous season. All national teams were first handled individually, but analysis revealed no significant differences between any national teams’ performance in fitness tests and therefore, all participants were split to only two groups: national team selected (n = 85, age 20 ± 3) and non-selected (n = 101, age 21 ± 4) players. Participants were included to selected players’ group if they had represented their country in senior- or youth-level between January 2022 and March 2023. Further, players were classified to the following playing positions: goalkeeper (GK), central-defender (CD), wide-defender (WD), central midfielder (CM), wide-midfielder (WM), and forward (FW). Groups’ descriptives are shown in table 1.

**TABLE 1:** Number of participants (percentage of group’s total n) from different playing positions in each group.

	Non-selected players	All national team selected players	National teams			
			Senior	U23	U19	U17
**Age, years**	**21 ± 4**	**20 ± 3**	24 ± 3	21 ± 2	18 ± 1	16 ± 0
**GK**	**16 (16%)**	**7 (8%)**	2 (13%)	0 (0%)	2 (7%)	3 (13%)
**CD**	**18 (18%)**	**14 (17%)**	1 (7%)	5 (28%)	6 (21%)	2 (8%)
**WD**	**18 (18%)**	**14 (17%)**	3 (20%)	1 (6%)	3 (11%)	7 (29%)
**CM**	**20 (20%)**	**17 (20%)**	3 (20%)	4 (22%)	4 (14%)	6 (25%)
**WM**	**15 (15%)**	**18 (21%)**	3 (20%)	3 (17%)	8 (29%)	4 (17%)
**FW**	**14 (14%)**	**15 (18%)**	3 (20%)	5 (28%)	5 (18%)	2 (8%)
**n total**	**101**	**85**	15	18	28	24

Senior = senior national-team. U23, U19 and U17 = under 23–17 national-teams. GK = goalkeeper, CD = central defender, WD = wide defender, CM = central midfielder, WM = wide midfielder and FW = forward.

Participants and parents of participants younger than 18-years were informed, verbally and in written form, of possible risks and discomforts associated with the study procedures and had the opportunity to discuss the study with the researchers. After that, participants signed an informed consent form. The study was approved by the Ethics Committee of the university (1375/13.00.04.00/2022) and conducted according to the Declaration of Helsinki (2013), except for registration in a database.

### Test procedures

In this cross-sectional study, all participants performed typical football performance tests: 30-meter sprint speed with 10-meter split time for linear speed, counter movement jump (CMJ) for lower-limb power and 1200-meter shuttle run test (1.2SRT, also known as the Bronco test) for endurance. These tests were selected because the Finnish Football Association has used these tests for several years in youth national team testing, as well as in youth player development program.

All tests were performed in each team’s or national team’s indoor training facilities. All tests were conducted by the same tester. National league teams were tested at the end of the pre-season (2–3 weeks before the start of the match-season, 2023) and national teams at the end of the match-season 2022. Testing time was different for national league and national team players because the only possibility to test the national teams was during international FIFA match windows in autumn 2022.

Tests were performed in an indoor football field selected by the teams. Before the tests, coaches were instructed to periodize either rest or a light training session the day prior to the tests. The teams’ fitness coaches oversaw the warm-up before the tests. A standardized warm-up protocol was introduced to coaches to follow (included 5 minutes jogging, activation and mobility exercises such as lunges, squats and single leg deadlifts and three sprints and jumps with increasing intensity), but coaches could modify in-line with their team’s habits. The test session started with sprint and jump tests and ended with the 1.2SRT. Total duration of the test session (warmup included) was 90–120 minutes depending on the number of players in the team.

The 30-meter sprint test with 10-meter split time, was performed on artificial turf wearing football shoes. For the test, players began 70 cm behind a photocell gate (Newtest Oy, Finland), which was one meter from the ground. Players performed three test trials, and the best time was used in analyses. Rest intervals between trials were three minutes at a minimum. Players’ maximal speed was measured from 30-meter sprints by radar (Stalker ATS II, Richardson, Texas, USA). Coefficient of variation (CV) percentage between the three trials were on average 1.2 ± 1.2% for 10-meter sprint time, 0.8 ± 0.8% for 30-meter sprint time and 1.2 ± 1.2% for maximal speed (by radar).

The CMJ test was performed on a hard surface wearing running shoes. Jump flight time was measured using an infrared mat (Custom built by the University of Jyväskylä) and flight time was converted to jump height using an equation: H = g × t^2^/8, where H is the jump height (m), t is the flight time of the jump (s), and g is the acceleration due to gravity (9.81 m · s-2). Players performed three test trials and the highest jump was used in final analyses. Rest intervals between trials were three minutes at a minimum. CV between three trials were on average 2.7 ± 1.6% for CMJ height.

The 1.2SRT was performed last on artificial turf with the entire team performing the test simultaneously. The test protocol was similar as described by Kelly & Wood [17]. As a warm-up, participants were instructed to jog the track once and then run it once at a self-determined speed in which they thought they will start the test. After the warm-up, participants had three minutes recovery before beginning the test. For the test, participants wore football shoes. 1.2SRT is not the most common test used in football, but strong correlations between this test and Yo-Yo intermittent recovery test 1 (Yo-Yo IR1) [18] and 30–15 intermittent fitness tests [17], have been shown. Also, high reliability of the 1.2SRT has been shown [19]. From 1.2SRT maximal aerobic speed (MAS) was calculated by the following formula: MAS = 1200 m / (test time in seconds – 20.3) [20]. Further the MAS value was used to calculate anaerobic speed reserve by subtracting a player’s MAS value from their maximal speed value.

### Statistical analyses

Statistical analyses were conducted using SPSS Statistics 28 (IBM, Armonk, NY). Results are reported as means ± standard deviation (SD). The significance level was set at p < 0.05. Data normality was confirmed by the Shapiro-Wilk test. One-way ANOVA revealed no significant differences between any national teams. Thus, all national team players were combined to a single group enabling playing-position specific (GK, CD, WD, CM, WM and FW) comparisons between national team selected and non-selected players, which were conducted by independent samples t-test. Effect sizes were calculated by using Hedges’ g. Effect sizes were classified using the following criteria: 0.2–0.5 small, 0.5–0.8 moderate, > 0.8 large.

Since position-specific analyses revealed significant differences between selected and non-selected players, possible differences between playing positions were analyzed individually for both groups (separately national team selected and non-selected players). Thus, possible differences between playing positions (GK, CD, WD, CM, WM and FW) specific to competition level were analyzed by Oneway ANOVA with Bonferroni post-hoc test.

## RESULTS

Table 2 shows differences between national team selected and non-selected players for each playing position. Selected CDs, WDs and CMs were faster in 10-meter sprinting and CMs and FWs in 30-meter sprinting compared to non-selected counterparts. Selected WMs reached higher CMJ height and FWs higher maximal speed compared to non-selected counterparts. Selected CMs, WMs and FWs reached higher MAS than non-selected counterparts. Also, selected CDs had higher anaerobic speed reserve than non-selected CDs.

**TABLE 2:** National-team selected (S) versus non-selected (NS) players’ physical qualities specific to playing position. Mean ± SD [n]. Significant (p < 0.05) and/or large effect sizes (*g* > 0.8) are emboldened.

		10-m sprint time (s)	30-m sprint time (s)	CMJ height (cm)	Maximal speed (m/s)	MAS (m/s)	Anaerobic speed reserve (m/s)
GK	S	1.96 ± 0.14 [7]	4.78 ± 0.29 [7]	33.0 ± 4.1 [7]	7.56 ± 0.36 [7]	3.89 ± 0.25 [7]	3.69 ± 0.25 [7]
	NS	2.00 ± 0.07 [15]	4.86 ± 0.18 [15]	29.5 ± 3.7 [15]	7.44 ± 0.33 [15]	3.81 ± 0.31 [15]	3.67 ± 0.5 [15]
	p	0.510	0.419	0.059	0.478	0.283	0.481
	*g*	-0.395	-0.377	**0.904**	0.331	0.278	0.023

CD	S	1.89 ± 0.06 [15]	4.64 ± 0.12 [15]	31.7 ± 2.0 [15]	7.72 ± 0.25 [15]	4.03 ± 0.25 [15]	3.69 ± 0.31 [15]
	NS	1.94 ± 0.07 [17]	4.71 ± 0.12 [17]	30.4 ± 3.0 [17]	7.58 ± 0.17 [17]	4.11 ± 0.22 [16	3.47 ± 0.19 [16
	p	**0.042**	0.079	0.071	0.081	0.204	**0.030**
	*g*	-0.751	-0.644	0.535	0.640	-0.302	**0.819**

WD	S	1.83 ± 0.06 [14	4.49 ± 0.14 [14	32.4 ± 3.6 [18	7.97 ± 0.33 [14	4.22 ± 0.19 [13	3.78 ± 0.36 [13
	NS	1.88 ± 0.05 [17]	4.55 ± 0.12 [17]	32.1 ± 4.4 [18	7.94 ± 0.28 [17]	4.19 ± 0.22 [16	3.78 ± 0.28 [16
	p	**0.014**	0.280	0.863	0.835	0.652	0.997
	g	**-0.939**	-0.455	0.064	0.078	0.168	0.001

CM	S	1.88 ± 0.06 [17]	4.62 ± 0.17 [17]	30.9 ± 3.9 [17]	7.72 ± 0.33 [17]	4.19 ± 0.28 [17]	3.53 ± 0.25 [17]
	NS	1.95 ± 0.08 [20	4.73 ± 0.14 [20	30.8 ± 2.9 [20	7.61 ± 0.22 [20	4.03 ± 0.25 [18	3.58 ± 0.33 [18
	p	**0.007**	**0.033**	0.483	0.254	**0.037**	0.304
	*g*	**-0.944**	-0.732	0.015	0.382	0.626	-0.175

WM	S	1.84 ± 0.08 [18	4.48 ± 0.19 [18	32.6 ± 2.9 [18	8.00 ± 0.36 [18	4.28 ± 0.19 [18	3.69 ± 0.36 [18
	NS	1.88 ± 0.06 [14	4.59 ± 0.11 [14	30.5 ± 3.5 [15]	7.81 ± 0.19 [14	4.08 ± 0.28 [13	3.72 ± 0.33 [13
	p	0.131	0.059	**0.048**	0.100	**0.018**	0.903
	*g*	-0.553	-0.700	0.720	0.619	**0.939**	-0.046

FW	S	1.89 ± 0.06 [15]	4.61 ± 0.14 [15]	32.4 ± 3.7 [15]	7.83 ± 0.28 [15]	4.25 ± 0.19 [14	3.61 ± 0.31 [14
	NS	1.94 ± 0.09 [12	4.73 ± 0.16 [12	30.5 ± 4.0 [14	7.58 ± 0.28 [12	3.94 ± 0.19 [12	3.64 ± 0.25 [12
	p	0.107	**0.047**	0.195	**0.023**	**< 0.001**	0.759
	*g*	-0.648	**-0.811**	0.493	**0.957**	**1.684**	-0.124

S = national-team selected players, NS = non-selected players. GK = goalkeeper, CD = central defender, WD = wide defender, CM = central midfielder, WM = wide midfielder and FW = forward.

**FIG. 1:**
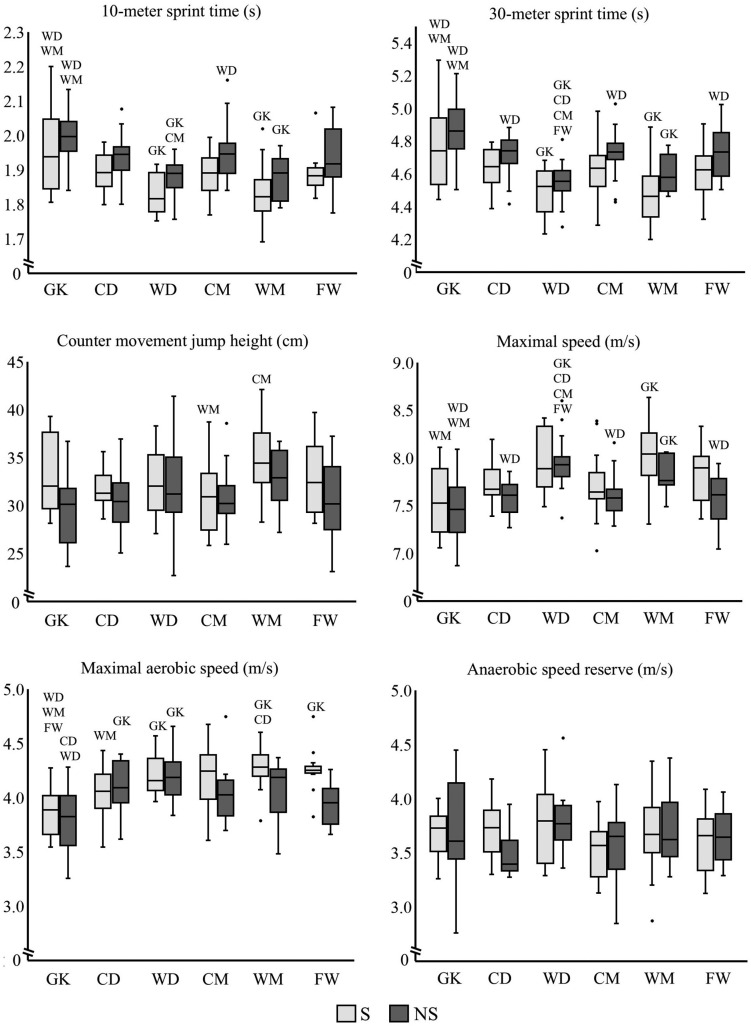
Test results of national-team selected (S) and non-selected players (NS) specific to playing position. Statistically significant (p < 0.05) within-group (S or NS) difference compared to GK = goalkeeper, CD = central-defender, WD = wide-defender, CM = centralmidfielder, WM = wide-midfielder and FW = forward.

Figure 1 shows differences between playing positions of selected and non-selected players in each test. Differences occurred mainly between GKs and outfield positions. Differences between outfield positions were selected WMs’ higher CMJ height compared to CMs, and selected WMs’ higher MAS compared to CDs, non-selected WDs’ faster 10-meter sprint time compared to CMs, and non-selected WDs’ faster 30-meter sprint time and higher maximal speed compared to CDs, CMs and FWs.

## DISCUSSION

The aim of this study was to investigate national team selected and non-selected players’ key physical qualities specific to playing positions. Results showed that physical qualities differentiate national team selected players from non-selected counterparts playing at a sub-elite level: selected GKs and WMs jumped higher, CDs, WDs and CMs were faster in 10-meter and CMs and FWs in 30-meter sprint tests, while CMs, WMs and FWs achieved higher MAS compared to non-selected counterparts. One novelty of the present study was to analyze between-position comparisons specific to competition level. There were differences in physical qualities mainly between GKs and outfield positions for both selected and non-selected groups. As expected, findings between outfield positions were different for selected and non-selected players. However, in both groups, players from wide-positions reached the higher performance in speed, power and/ or endurance tests compared to central-positions. Selected WMs reached higher CMJ height compared to CMs and selected WMs had higher MAS compared to CDs while non-selected WDs were faster for 30-meter sprint time and reached higher maximal speed compared to CDs, CMs and FWs.

There were no differences in test results between any national teams. This finding contradicts previous studies, which have shown that senior national team players have performed better in endurance, speed and strength or power tests compared to U17 national team players [2, 3]. Also, another study with a large sample showed that female football players’ physical qualities improve until around the age of 25-years, but it is important to notice that development is not linear [21]. Further, a recent study from Sweeney et al., [22] showed that female players selected to U15 and U16 national teams are biologically more mature compared to the general population, which demonstrates that national team selected players are a specific group. This could, at least partially, explain why there were no differences in physical qualities between national teams in the present study. Also, performance in all tests used in the present study required moving the body’s mass. Typically, senior players are heavier than junior players [2, 3], which can favor junior players in tests where body mass needs to be transported. In the football match on the other hand, some actions benefit from greater absolute force, for example maximal back squat load, which is associated to tackling success [23] and therefore, the tests used in the present study do not cover all physical qualities needed to perform in a football match. Finally, it must be acknowledged that players are selected to the national team based on the coaches’ holistic evaluation rather than a sole focus on physical qualities, which may explain the findings of the present study.

Previous study [6] suggested that physical qualities generally do not distinguish between performance levels in elite-level football, but the present study shows that physical qualities differentiate national team selected players from non-selected sub-elite-level counterparts. Selected players were significantly faster in either 10- or 30-meter sprint tests compared to non-selected players in all outfield positions except in WMs. However, there was a trend with a moderate effect size (p = 0.059, g = –0.70) in WMs’ 30-meter sprint time in favor of selected players. Therefore, it seems that sprint speed is a crucial factor to differentiate national team selected players from non-selected sub-elite level counterparts regardless of playing position. This is logical because high-intensity running distance is a determining factor for performing at international-level [8] and sprint speed is one key factor predicting match high-intensity (> 19 km/h) running distance [7]. Since sprint speed is such an important quality for female football players, especially coaches of sub-elite level players are encouraged to improve it through sprint [24], strength or plyometric training [25].

Selected CMs, WMs and FWs had higher MAS compared to non-selected counterparts. Also, this finding is logical because performance in Yo-Yo IR1 (which has a strong correlation to 1.2SRT used in present study [18]) was shown to relate to match high-intensity running distance [26] and therefore, to competition level [8]. Coaches should aim to improve MAS in non-selected CMs, WMs and FWs by small-sided games or high-intensity interval training to help players to reach their potential [27]. There were no significant differences between selected and non-selected GKs, but in CMJ height there was a trend with a large effect size (p = 0.059, g = 0.904) to favor selected GKs. Considering GKs’ role, this finding is logical because good vertical jumping is important for GKs, as they are often required to leap vertically to catch or deflect the ball [28]. Plyometric training has shown to be an effective training method for female football players to improve vertical jump height [25] and, therefore, it is recommended. To improve vertical jump height, horizontally orientated plyometrics can be recommended, because those are as effective as vertical plyometrics to improve vertical jumping performance, but simultaneously superior to improve horizontal jumping performance [29], at least in male populations.

Similar study from USA’s NWSL found significant differences between national team selected and non-selected players only for defensive midfielders’ sprint test times [6]. NWSL is one of the top leagues in the world and all players are elite-level professionals regardless of national team status [16]. For example, comparison of sprint test times from NWSL non-selected players and the present study’s selected players demonstrates the high-level attained in NWSL non-selected players. NWSL non-selected players’ average sprint time over 30-meters was better than the present study’s selected players in certain positions (for example CDs 4.46 s vs. 4.64 s and FWs 4.41 s vs. 4.61 s) [6]. Therefore, it seems plausible that a high-level of physical qualities are required to reach the elite-level, which may then be observed by the minor differences in physical qualities between national team selected and non-selected NWSL players. Once the elite-level is achieved, it could be argued that other variables (such as technical or tactical) are more determinative factors to explain national team status. Instead, playing in sub-elite level is possible without top physical qualities, which could explain the significant differences between national team selected players and sub-elite level non-selected players in present study.

Since there were differences between selected and non-selected players specific to playing position, between-position comparisons were performed for both groups separately. Comparing groups separately is also relevant based on findings from previous studies, which have shown differences in physical qualities between outfield positions for elite players [5], but not for national level players [15]. In both groups there were differences mainly between GKs and outfield positions as expected [4, 13, 15]. The reason is naturally the completely different match demands for GKs compared to outfield positions [11]. As expected, differences in physical qualities between outfield positions varied between groups [5, 15]. However, in both groups’, players from wide-positions reached the higher performance in speed, power and/ or endurance test compared to central-positions.

Selected WMs achieved higher MAS compared to CDs. This was expected, because Bradley et al. [5] showed similar findings from the Yo-Yo IR1 endurance test in elite-level players. Since WMs cover higher total- and high-intensity running distance than CDs in elite-level match-play [10], it could be that playing a specific position with certain demands lead to training adaptations over the years or players with high aerobic capacity are selected to play wide-midfield and players with lower aerobic capacity to central defense. Selected WMs also achieved higher CMJ height compared to CMs. This finding highlights the mixed demands of selected WMs. They have to be simultaneously powerful and have high aerobic capacity, which has to be considered in their training periodization to avoid the interference effect.

Contrasting findings from Vagle et al., [15], there were also differences between non-selected players. As in selected players, non-selected players from wide positions performed better compared to players from central positions. Non-selected WDs performed better in 30-meter sprinting and reached higher maximal speed than CDs, CMs and FWs. Vagle et al., [15] concluded that different playing positions need to be somewhat equal in physical qualities for the given position to match the opponent opposite (i.e., attacker vs defender). According to this theory, perhaps coaches in Finland’s premier national league prefer fast WDs to improve their teams’ defensive line, which could explain the finding. Nevertheless, it seems that position-specific demands/ roles may differ between competition levels, which must be considered in future studies.

The biggest strength of this study was a high number (n = 186) of participants [2, 3, 14, 15], which allowed comparisons between competition levels specific to playing position and between-position comparisons individually for both selected and non-selected players, which was a novel approach. The CV%s between trials in sprint and CMJ tests (10-meter 1.2%, 30-meter 0.8% and CMJ 2.7%) showed that variation between test trials were similar or lower compared to a previous study [21] (5-meter 1.2%, 30-meter 3.9% and CMJ 3.9%) in female players, which confirms reliability and the appropriateness of the tests used.

The first limitation of this study was that national teams and national league teams were tested in different phases of the season because national teams had to be tested during international FIFA match windows. Thus, there is potential that fitness levels had changed during the course of the season creating an issue in making direct comparisons between groups. Nevertheless, our currently unpublished data from 158 Finland’s premier national league players showed that physical qualities remained stable during the in-season, 2023. Further, previous studies have shown similar no change in endurance capacity [30–33], in lower-limb strength and power [31–33] and in speed or change of direction [33] in female football players across the in-season. Therefore, we assert that the effect of different testing time between groups was not a major limitation here. The second limitation was that players were classified to groups based on their self-reported playing position. Since players’ role in the same position can vary a lot depending on the team’s tactics, more sophisticated classification is needed to investigate differences of different tactical roles in future. The third limitation was that players’ anthropometry or body composition were not reported, because of inadequate data. Performance in all tests used in the present study required moving the body’s mass and, thus, information about anthropometry and body composition would have given extra information to interpret differences between selected and non-selected players and between-positions. However, a similar previous study from Scott et al. [6] did not report players’ anthropometry either and, thus, it should be investigated in the future.

### Practical implications

Sprint time seems to be a differentiative factor between players selected and those not selected for national teams in all outfield positions. Further, non-selected wide-defenders were faster than counterparts from central-positions. Thus, it is imperative that speed qualities are trained, especially during development, so that it does not restrict players’ competition level nor playing position. Similarly, lower limb power was a distinctive feature of selected players from wide-midfield positions. Coaches are advised to focus training above current practice for speed and power. However, speed is traditionally difficult to make considerable improvements through training. Thus, players that have high-level football proficiency should recognize whether they naturally have the speed demanded of wide-playing positions, if not they are recommended to specialize for central-positions. Second, maximal aerobic running speed distinguished between selected and non-selected players in central- and wide-midfielders and forwards. It also differentiates wide-midfielders from central-defenders in national team selected players. Current practice appears to be insufficient for aerobic performance in non-selected midfielders and forwards and, therefore, their training load and/or intensity should be increased progressively to improve aerobic qualities.

## CONCLUSIONS

The present study showed that there are differences between national team selected and non-selected female football players’ physical qualities. Previous studies suggested that physical qualities generally do not distinguish between performance levels in elite-level football, but the present study shows that physical qualities differentiate national team selected players from sub-elite level counterparts. In particular, performance in speed tests differentiate selected from non-selected players of the same outfield playing position(s), thus, speed appears critical which may limit player’s progress. Non-selected wide-defenders were faster in 30-meter sprint test than central defenders, central-midfielders and forwards, which highlights the importance of speed qualities especially over longer distances for this position in non-selected players. Instead, selected players’ wide-midfielders reached higher jump height than central-midfielders and higher maximal aerobic speed compared to central-defenders. These findings emphasize mixed demands of national team selected wide-midfielders when high aerobic capacity and lower-limb power are required simultaneously. Since between-position differences varied between selected and non-selected players it seems that position-specific demands/ roles may differ between competition levels, which must be considered in future studies.
